# Complement Inhibition as a Proposed Neuroprotective Strategy following Cardiac Arrest

**DOI:** 10.1155/2009/124384

**Published:** 2010-01-26

**Authors:** Brad E. Zacharia, Zachary L. Hickman, Bartosz T. Grobelny, Peter A. DeRosa, Andrew F. Ducruet, E. Sander Connolly

**Affiliations:** Department of Neurological Surgery, Columbia University, 710 W. 168th Street, New York, NY 10032, USA

## Abstract

Out-of-hospital cardiac arrest (OHCA) is a devastating disease process with neurological injury accounting for a disproportionate amount of the morbidity and mortality following return of spontaneous circulation. A dearth of effective treatment strategies exists for global cerebral ischemia-reperfusion (GCI/R) injury following successful resuscitation from OHCA. Emerging preclinical as well as recent human clinical evidence suggests that activation of the complement cascade plays a critical role in the pathogenesis of GCI/R injury following OHCA. In addition, it is well established that complement inhibition improves outcome in both global and focal models of brain ischemia. Due to the profound impact of GCI/R injury following OHCA, and the relative lack of effective neuroprotective strategies for this pathologic process, complement inhibition provides an exciting opportunity to augment existing treatments to improve patient outcomes. To this end, this paper will explore the pathophysiology of complement-mediated GCI/R injury following OHCA.

## 1. Introduction

A dearth of effective treatment strategies exist for global cerebral ischemia-reperfusion (GCI/R) injury following successful resuscitation from out-of-hospital cardiac arrest (OHCA). OHCA is a devastating disease process with neurological injury accounting for a disproportionate amount of the morbidity and mortality following return of spontaneous circulation (ROSC). The incidence of OHCA in industrial countries ranges from 0.04 to 0.13% of the total population per year, and only 11–48% of patients admitted to the hospital are discharged in good neurologic condition. At present, therapeutic hypothermia is the only neuroprotective strategy shown to be effective in well-controlled, prospective trials of OHCA. 

Complement, an important component of the innate immune system, is known to play a central deleterious role in multiple diverse disease processes. Eculizumab, a monoclonal C5-antibody, is currently being used to treat paroxysmal nocturnal hematuria, and pexelizumab, also a C5-antibody, has been studied as adjunctive therapy in ischemic heart disease [1, 2]. Emerging preclinical as well as recent human clinical evidence also suggests that activation of the complement cascade plays a critical role in the pathophysiology of GCI/R injury following OHCA [3]. In addition, it is well established that complement inhibition improves outcome in both global and focal models of brain ischemia [4–8]. Due to the profound impact of GCI/R injury following OHCA, and the relative lack of effective neuroprotective strategies for this pathologic process, complement inhibition provides an exciting opportunity to augment existing treatment to improve patient outcomes. To this end, this paper will explore the pathophysiology of complement-mediated GCI/R injury following OHCA.

## 2. The Impact of out-of-Hospital Cardiac Arrest

Heart disease remains the leading cause of mortality in the United States, and most frequently presents as sudden out-of-hospital cardiac arrest (OHCA) [9]. Recent sources indicate that approximately 166,000–310,000 Americans experience an OHCA per year [10], with a variable number undergoing resuscitation. Given this wide variability in reported incidence, survival and outcome following OHCA are difficult to accurately assess [9]. A multitude of factors has been shown to influence survival following OHCA, including demographic, clinical, and treatment factors [11–19] and attempts at influencing those factors that are modifiable stand to potentially improve outcome. Brain injury from global cerebral ischemia-reperfusion is a major factor limiting the survival and functional recovery of patients after resuscitation from OHCA. In most cases, survivors have significant impairment of consciousness and may eventually progress to a persistent vegetative state [14]. The acute and long-term care for these survivors comes with a staggering cost to healthcare systems, patients' families, and society as a whole [20, 21].

Historically, survival following OHCA has been poor [22, 23], and although the morbidity and mortality of most cardiovascular diseases have declined over the last 30 years [10] there has been little improvement in survival post-OHCA [24, 25]. Even in those patients that are resuscitated and survive the initial insult, crippling neurological deficits from the global cerebral ischemia experienced during the arrest are frequent. Survival for patients with an OHCA has been reported between 1 to 31% with significant regional variation [9, 10]. In fact, a recent large, prospective, multicenter observational study throughout North America demonstrated that only 7.9% of treated cardiac arrest patients survive until discharge [9]. Multiple out-of-hospital factors, including bystander cardiopulmonary resuscitation (CPR), time to defibrillation, and EMS experience have all been associated with differences in survival after resuscitation [11, 26–28], yet the effect of hospital-based postresuscitation care on outcomes has been largely ignored. 

With advances in EMS defibrillation programs, an increasing number of patients survive to hospital admission after OHCA. Of those patients, only 11–48% will be discharged from the hospital with good neurologic outcome [29]. Recently, in-hospital therapeutic hypothermia was established as an option to improve neurologic outcome after OHCA [30, 31]. Another study suggested that hemofiltration to reduce inflammation after OHCA confers additional survival benefit, but this remains unproven [32].

## 3. The Inflammatory Response following OHCA

With arrest of systemic and cerebral blood flow for greater than 5 minutes, a series of events is initiated, inciting an inflammatory cascade resulting in significant cerebral injury [9, 10, 23, 33]. During and after cardiopulmonary resuscitation, blood coagulation, platelet activation with formation of thromboxane A_2_, and an alteration of soluble E-selectin (sE-selectin) and P-selectin (sP-selectin) have been described [34–36]. A postresuscitation syndrome, characterized by hyperthermia, hypotension, and multiple organ failure is likely the clinical expression of whole-body ischemia-reperfusion (I/R) injury occurring after return of spontaneous circulation [32, 37]. This syndrome is associated with both complement activation and an intense increase of various inflammatory mediators (IL-1, IL-6, IL-8, and IL-10) as early as 3 hours after cardiac arrest, and thus affords potential targets for new treatments [37].

## 4. Complement and Cerebral Ischemia

The complement system, a phylogenetically conserved component of the innate immune system, has been shown to be significantly involved in ischemia-reperfusion injury in multiple organ systems, including the central nervous system (CNS). The complement cascade contains more than 20 proteins and is involved in inflammation, opsonization, and cytolysis in a wide range of diseases [38–44]. Complement activation refers to the process of complement cascade initiation and execution, which results in the production of inflammatory mediators (C3a, C5a), the opsonization of cells with components (C3b) for recognition and phagocytosis by macrophages, and the formation of lethal membrane attack complexes (MAC, C5b-9) on target membranes [45].

**Figure 1:**
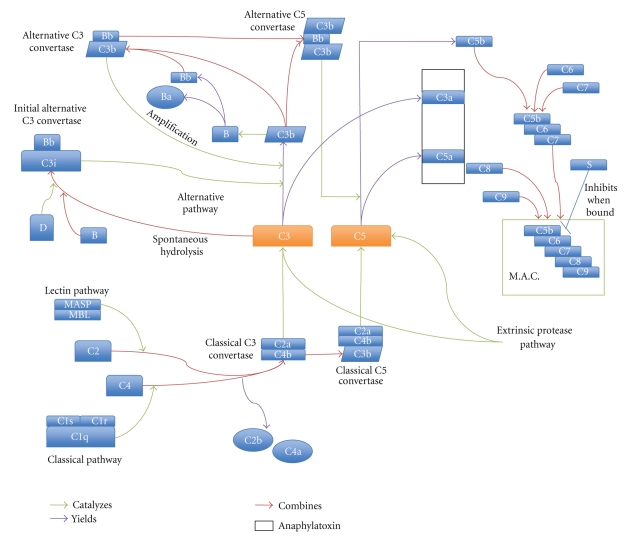
Complement cascade.

Complement may be activated via one of several ways—the classical, alternative, and lectin pathways. The classical pathway is initiated by the binding of C1q to antibody-antigen complexes while the lectin pathway begins when pathogen-associated molecules become bound by lectin proteins, such as MBL. Both of these pathways then catalyze the cleavage of the C2 and C4 complement components which form a C3 convertase. Cleaved C3 then is incorporated into a C5 convertase which leads to the cleavage of C5 and the subsequent assembly of the MAC. The alternative pathway, however, relies on spontaneous hydrolysis of C3 to form C3 and C5 convertases which lead to the formation of the MAC. Also, there are several proteolytic enzymes such as elastases, kallikrein, and thrombin, which can cleave C3 or C5, directly [46–48] (Figure 1).

With regards to the central nervous system, activation of complement has been implicated in the pathophysiology of several distinct diseases, including multiple sclerosis [49], development of vasospasm following aneurysmal subarachnoid hemorrhage [50, 51], as well as stroke [5, 6, 52]. 

Increasing evidence demonstrates that cerebral ischemia is followed by an acute systemic inflammatory response of the host. The complement system plays an essential and specific role in most pathological inflammatory events (upregulation of adhesion molecules, neutrophil activation, chemotaxis, expression of IL-8, and MCP-1 by endothelial cells) which occur shortly after the ischemic insult [53]. The essential role of complement activation in both microvascular failure and direct neuronal cell death was demonstrated in experimental animal models of permanent and transient middle cerebral artery occlusion (MCAO) through an increase in the expression of C3a and C5a receptors and presence of C1q and C3 in the core of the infarct [54, 55]. Our group subsequently demonstrated significant reduction of both infarct volume and oxidative stress after transient MCAO in C3 knock-out mice [6]. Furthermore, treatment of wild- type mice with C3a receptor antagonist (C3aRA) resulted in reduced stroke volumes and improved neurologic function following transient cerebral ischemia [5, 6]. Recently, Arumugam et al. reported that C5-deficient mice were significantly protected from I/R injury compared to wild-type littermates, and our group supported this data by demonstrating that C5a receptor antagonism reduces infarction volume in mice [56, 57]. In further rodent experiments, broad spectrum complement inhibitor cobra venom factor (CVF) [8, 56, 58], intravenous immunoglobulin [56], C1-inhibitor [59, 60], and sCrry [61] have all been shown to be protective against experimental cerebral ischemia via complement inhibition.

Studies investigating complement activation following human cerebral ischemia have been more limited. Pederson et al. [54] and Mocco et al. [52] reported elevations of SC5b-9 and C3a, respectively, in human stroke patients. In a recent report, Szeplaki et al. further contributed to the body of knowledge regarding complement activation in cerebral ischemia by demonstrating early cleavage of multiple complement components and an association between degree of complement activation and clinical severity and unfavorable outcome [53]. There is also growing recognition that complement activation contributes to the pathogenesis of global hypoxic-ischemic (HI) injury in both rodent models and in human neonates. Circulating C3 is depleted following birth asphyxia, and it was recently demonstrated that pretreatment with CVF significantly reduced brain infarcts in p7 rats subjected to hypoxia-ischemia [45]. Additionally, Hedtjarn et al. noted that a number of genes involved in the complement system were induced by HI in the immature brain, including C3a receptor, C5a receptor, and C1q. Schultz et al. [62] also demonstrated significant upregulation of C9 in human infants who developed moderate to severe hypoxic-ischemic encephalopathy and experimental animal work by our group [7] demonstrated upregulation of C1q and C3 after an hypoxic-ischemic insult in mice. We further demonstrated significant protection in C1q knockout mice compared with wild-type littermates, highlighting the central role of the classical complement cascade in global cerebral hypoxic-ischemic injury. However distinct neonatal physiology may be, similar results have been found in studies of mature animals in regards to complement activation in the setting of transient global cerebral ischemia. Schafer et al. demonstrated early and widespread upregulation of C1q expression in brain microglia and secretion of functionally active C1q into the CSF in response to experimentally induced global cerebral ischemia in rats [63].

## 5. Ischemia/Reperfusion Injury following out-of-Hospital Cardiac Arrest

Although reperfusion is essential for ultimate tissue survival, it may exacerbate cerebral injury and thus presents a treatment paradox [64]. As demonstrated above, activation of complement plays a critical role in ischemia-reperfusion injury leading to increased vascular permeability, activation of the coagulation cascade, free-radical production, and direct tissue damage [3, 65, 66]. Several models of ischemia-reperfusion injury have implicated different complement activation pathways in this pathology, though less is known how these pathways are initiated in ischemia-reperfusion [7, 63, 67–71] (Table 1). Cardiac arrest and resuscitation represent a whole-body I/R syndrome [3], yet the role of complement in the pathophysiology of OHCA has only recently begun to be elucidated. Pretreatment with the proteinase inhibitor aprotinin and with heparin, both of which reduce complement activation [72], increases survival after cardiac arrest in rabbits [73]. In a swine model, aprotinin enhanced the recovery of cerebral energy metabolism after deep hypothermic circulatory arrest [74]. In the sole study looking at complement levels following OHCA in humans, Bottiger et al. demonstrated significant systemic upregulation of complement components C3a and SC5b-9 during cardiopulmonary resuscitation and early reperfusion after cardiac arrest [3]. The question remains, however, whether complement activation after cardiac arrest in humans is mechanistically involved with disease pathogenesis and impacts neurologic outcome following OHCA [3].

## 6. Current Management of Global Cerebral Ischemia-Reperfusion Injury

Recently, two trials demonstrated that induced hypothermia confers a neuroprotective effect in patients who were resuscitated from cardiac arrest [30, 31]. Clinical and experimental results demonstrate a multifactorial neuroprotective effect of hypothermia during and after an ischemic insult by simultaneous suppression of several damaging pathways [29]. This has since become standard-of-care for select OHCA patients in centers where therapeutic hypothermia is offered. The proposed mechanism of hypothermia's protective effect has largely been attributed to its preservation of metabolic substrates, alteration of cerebral blood flow, and prevention of excitatory amino acid accumulation [75, 76]. Other work, however, has shown that the profound effects of hypothermia on ameliorating cerebral injury are not fully explained by these factors. Some have thus proposed that mild hypothermia also has anti-inflammatory effects, although there are conflicting results in the literature [76–78]. Recently, in an elegantly designed rodent study, Callaway et al. demonstrated that hypothermia following cardiac arrest does not alter serum inflammatory markers, including TNF-alpha, MCP-1, IL-2, IL-9, and IL-10, suggesting that the beneficial effects of hypothermia do not arise from attenuation of the inflammatory response [79]. Nevertheless, they confirmed a significant acute upregulation of inflammatory markers following ROSC. An alternate explanation is that the cerebral inflammatory response following cardiac arrest may not be accurately reflected in serum measurements of inflammatory biomarkers [79, 80]. The findings detailed above raise the possibility of additional therapeutic benefit from targeted anticomplement and anti-inflammatory strategies combined with hypothermia in the setting of OHCA.

## 7. Conclusion

While there exists little data concerning the activation of complement in humans following OHCA, the importance of complement activation, and in particular C3, has been demonstrated repeatedly in the pathogenesis of ischemia-reperfusion injury in both humans and various animal models [50, 51]. Selective complement inhibition, particularly of C3, is therefore an attractive strategy for global cerebral ischemia-reperfusion injury following OHCA, and may thereby improve long-term outcomes. While complement activation may be deleterious in the acute setting, it has been shown to be involved in modulation of neurogenesis, as well as the orderly clearance of apoptotic cell bodies, and therefore may have long-term beneficial effects [81]. As such, novel compounds that are able to reversibly inhibit downstream complement components, such as C3 and C3a, in the acute postischemic period may offer the best chance for a therapeutic benefit in human OHCA patients.

## Figures and Tables

**Table 1:** Complement pathways in ischemia-reperfusion injury.

Pathway	Type of I-R Injury	Study
Classical	Brain	Schäfer et al. [63], Ten et al. [7]
	Skeletal muscle	Weiser et al. [71]

Alternative	Gastrointestinal	Hart et al. [69]
	Renal	Thurman et al. [67], Zhou et al. [70]

Lectin	Myocardial	Jordan et al. [68]
	Gastrointestinal	Hart et al. [69]
	Skeletal Muscle	Weiser et al. [71]

I-R: ischemia-reperfusion.
